# Association Between Hearing Amplification, Satisfaction with Healthcare Communication, and Quality of Life Among Older Adults with Hearing Loss

**DOI:** 10.3390/healthcare14213743

**Published:** 2026-11-03

**Authors:** Djordje Zdravkovic, Ivana Milojevic, Andjela Gogic, Jovana Radovanovic Selakovic, Viktor Selakovic, Snezana Radovanovic, Milos Stepovic, Olgica Mihaljevic, Jasmina Stojanovic, Strahinja Krsmanovic, Katarina Janicijevic, Sanja Ilic, Stevan Matic, Darija Gvozdenovic, Aleksandra Dimitrijevic, Ivana Simic Vukomanovic

**Affiliations:** 1 Department of Otorhinolaryngology, Health Center “Sveti Luka” Smederevo, 11300 Smederevo, Serbia; drzdravkovic@hotmail.com (D.Z.); milojevic86ivana@gmail.com (I.M.); 2 Department of Medical Statistics and Informatics, Faculty of Medical Sciences, University of Kragujevac, 34000 Kragujevac, Serbia; andjelica97@hotmail.com; 3 Department of Epidemiology, Faculty of Medical Sciences, University of Kragujevac, 34000 Kragujevac, Serbia; radovanovicjovanaaa@gmail.com; 4 University Clinical Center Kragujevac, University of Kragujevac, 34000 Kragujevac, Serbia; 5 Department of Communication Skills, Ethics, and Psychology, Faculty of Medical Sciences, University of Kragujevac, 34000 Kragujevac, Serbia; selakovicviktor@gmail.com; 6 Department of Social Medicine, Faculty of Medical Sciences, University of Kragujevac, 34000 Kragujevac, Serbia; jovanarad@yahoo.com (S.R.); kaja.andreja@yahoo.com (K.J.); sanjailic84@yahoo.com (S.I.); stmatic769@gmail.com (S.M.); drivanasimic@gmail.com (I.S.V.); 7 Institute of Public Health Kragujevac, 34000 Kragujevac, Serbia; darijasapic@gmail.com (D.G.); alexandrarad84@gmail.com (A.D.); 8 Department of Anatomy, Faculty of Medical Sciences, University of Kragujevac, 34000 Kragujevac, Serbia; 9 Department of Pathophysiology, Faculty of Medical Sciences, University of Kragujevac, 34000 Kragujevac, Serbia; vrndic07@yahoo.com; 10 Department of Otorhinolaryngology, Faculty of Medical Sciences, University of Kragujevac, 34000 Kragujevac, Serbia; fonijatarkg@gmail.com (J.S.); skrsmanovic8@gmail.com (S.K.); 11 Clinic for Otorhinolaryngology, University Clinical Center Kragujevac, 34000 Kragujevac, Serbia

**Keywords:** hearing amplification, hearing aids, quality of life, communication with healthcare professionals, hearing handicap, WHOQOL-BREF, IOI-HA, HHIE

## Abstract

Background and Objectives: This study examined changes in hearing handicap and quality of life following hearing amplification in older adults and associations of hearing aid outcomes and healthcare communication satisfaction with post-amplification quality of life. Materials and Methods: This prospective, multicenter study included 117 adults aged over 60 years with bilateral severe sensorineural hearing loss and no previous hearing aid use. Hearing handicap, quality of life, hearing aid outcomes, and healthcare communication satisfaction were assessed using the Hearing Handicap Inventory for the Elderly (HHIE), WHOQOL-BREF, International Outcome Inventory for Hearing Aids (IOI-HA), and a 12-item communication satisfaction measure, respectively. Results: Hearing amplification was associated with significantly reduced hearing handicap and improved quality of life across all WHOQOL-BREF domains (*p* < 0.01). Healthcare communication satisfaction also improved significantly (*p* < 0.001). Better hearing aid outcomes were associated with higher post-amplification quality of life across all domains, remaining independently associated after adjustment for baseline quality of life and participant characteristics. Greater healthcare communication satisfaction was positively associated with all quality-of-life domains but remained independently associated only with social relationships after adjustment. Conclusions: Hearing amplification was associated with reduced hearing handicap, improved quality of life, and greater healthcare communication satisfaction. Better hearing aid outcomes were independently associated with higher quality of life across all domains, whereas communication satisfaction was independently associated only with social relationships. This finding warrants caution given the lower internal consistency of that domain.

## 1. Introduction

Hearing loss is one of the most common health conditions affecting older adults and, due to population ageing, is expected to become an increasingly important public health challenge [1]. According to estimates by the World Health Organisation, approximately 430 million people worldwide currently experience disabling hearing loss. The global burden of this condition is expected to increase substantially, with nearly 2.5 billion people projected to have some degree of hearing loss by 2050, while more than 700 million will require appropriate hearing rehabilitation [2]. These projections highlight the growing public health importance of hearing loss and the need for its systematic integration into healthcare planning. Reducing the future burden on healthcare systems requires a comprehensive approach that includes raising public awareness, prevention, timely detection of hearing loss, and improving access to affordable hearing aids and other forms of rehabilitation [2].

Assessment of quality of life is an important component of a comprehensive approach to individuals with hearing loss, as it provides insight into the consequences of auditory impairment on everyday functioning and patients’ subjective well-being [3]. Hearing loss may be associated with adverse psychological and social outcomes, including depressive and anxiety symptoms, feelings of loneliness, and reduced social participation, all of which may negatively affect quality of life [4,5]. The consequences of hearing loss may also extend to broader social and economic aspects of an individual’s functioning, including educational opportunities, employment, and productivity [6].

Effective communication between patients and healthcare professionals is an important component of high-quality healthcare, as it may contribute to greater patient satisfaction, better cooperation during treatment, and more favourable health outcomes [7]. Hearing loss can represent a substantial barrier to effective communication in healthcare settings, potentially adversely affecting patients’ experiences and satisfaction with the care they receive [8,9]. Communication difficulties, insufficient cultural competence among healthcare professionals, and limited availability of appropriate communication support may negatively affect the experiences of individuals with hearing loss when accessing healthcare services, increasing feelings of exclusion, unequal treatment, and discrimination [10]. Understanding health-related information may also be challenging for individuals with hearing loss due to the use of complex medical terminology and inadequately adapted communication approaches. The use of simpler language, verification of patients’ understanding, and written or visual materials may represent effective strategies for improving communication [11,12].

The use of hearing aids has been associated with improvements in both general quality of life and hearing-specific aspects of quality of life that are directly affected by hearing impairment. The most pronounced positive effects have been observed when quality of life is assessed using instruments specifically designed for individuals with hearing loss [13]. Hearing aid use may also help reduce the psychological, social, and emotional consequences of hearing loss, thereby contributing to improved everyday quality of life among users [13,14]. Evidence from a randomized controlled trial further supports the benefits of conventional hearing-aid provision, demonstrating improvements in hearing-related quality of life and reductions in self-perceived hearing handicap following hearing-aid use [15].

Hearing rehabilitation, however, encompasses a broader range of approaches, including conventional hearing aids, personal sound amplification devices, communication-focused interventions, and other rehabilitative strategies. These approaches differ in their components and intended effects, and evidence obtained for one form of intervention should not necessarily be extrapolated to another. Accordingly, the present study focuses specifically on conventional hearing-aid amplification, while evidence from other hearing-assistance and rehabilitation approaches provides broader contextual rather than directly comparable evidence.

Conceptually, hearing amplification, hearing-aid outcomes, communication experiences, social participation, and quality of life may be understood as interrelated components of the broader hearing-rehabilitation experience. Improved access to auditory information may reduce perceived hearing handicap and facilitate communication, while favourable hearing-aid outcomes, including perceived benefit and fewer activity and participation restrictions, may be associated with better social functioning and quality of life [16,17]. Within healthcare settings, improved access to spoken communication may also be associated with more favourable communication experiences with healthcare professionals [8,9,10,11,12]. These relationships provide a conceptual framework for the present study but are not intended to represent a causal or mediational model. In particular, satisfaction with healthcare communication was considered a correlate of quality of life rather than a mechanism through which hearing amplification affects quality of life.

Although previous studies have examined the effects of hearing aid use on hearing-related outcomes and quality of life, these aspects have often been investigated separately from patients’ experiences of communication within healthcare settings. Evidence remains limited regarding the simultaneous assessment of hearing aid outcomes, satisfaction with communication with healthcare professionals, and multidimensional quality of life among older adults following hearing amplification. The present study addresses this gap by simultaneously evaluating changes in self-perceived hearing handicap and quality of life following hearing amplification, while also examining their associations with hearing aid outcomes and satisfaction with healthcare communication. This integrated approach may provide a more comprehensive understanding of the benefits of hearing rehabilitation beyond improvements in hearing alone.

Therefore, this study aimed to examine changes in self-perceived hearing handicap and quality of life following hearing amplification among older adults with hearing loss, as well as the associations of hearing aid outcomes and satisfaction with communication with healthcare professionals with quality of life following hearing amplification.

Accordingly, we hypothesized that HHIE scores would be lower and WHOQOL-BREF scores higher following hearing-aid fitting compared with baseline. We further hypothesized that higher IOI-HA scores would be associated with higher post-amplification quality of life after adjustment for baseline quality of life and relevant participant characteristics, and that greater satisfaction with healthcare communication would be particularly associated with the social relationships domain.

## 2. Materials and Methods

### 2.1. Study Design and Setting

The study was designed as a prospective, multicenter, analytical study. Participant recruitment was conducted from July 2025 to June 2026. Potentially eligible patients were consecutively assessed during the recruitment period.

The study was conducted over a 12-month period, from July 2025 to June 2026, after obtaining approval from the Ethics Committees of the participating healthcare institutions. Patients were recruited from the otorhinolaryngology specialist outpatient clinics of the following healthcare institutions: “Sveti Luka” Health Centre, Smederevo; “Stefan Visoki” General Hospital, Smederevska Palanka, and Požarevac General Hospital. Of the 117 participants included in the final analytical sample, 90 (76.9%) were recruited at the “Sveti Luka” Health Centre in Smederevo, 19 (16.2%) at the “Stefan Visoki” General Hospital in Smederevska Palanka, and 8 (6.8%) at Požarevac General Hospital. Participants were enrolled only after receiving comprehensive information about the study and providing written informed consent.

The research team was required to provide each participant with a printed information sheet detailing the purpose and objectives of the study, participants’ rights, and information on how and where to submit complaints or concerns in the event of a perceived violation of their rights. The study was conducted in accordance with established ethical principles, international ethical standards, including the Declaration of Helsinki [18], and the relevant legislation of the Republic of Serbia.

### 2.2. Study Population and Sample Size

During the recruitment and follow-up process, 139 potentially eligible patients were assessed. Of these, 117 completed the follow-up assessment, met the predefined criteria for inclusion in the final analytical sample, and were included in the analyses. Of the remaining 22 patients, eight did not obtain a hearing aid, two died after hearing amplification before the follow-up assessment, ten did not return the follow-up questionnaires and did not attend follow-up visits, and two reported insufficient hearing-aid use (approximately 1–2 h per day) and therefore did not meet the predefined criterion for effective hearing-aid use. The final analytical sample therefore comprised 117 participants.

The original sample-size planning did not include a formally prespecified single primary endpoint, an expected pre-post mean difference and standard deviation of the paired differences, or a predefined attrition rate from which a statistically derived target sample size could be calculated. Therefore, rather than retrospectively introducing assumptions that were not specified before recruitment, the statistical properties of the achieved sample were characterized using sensitivity analyses in G*Power 3.1.

For the pre-post analyses, a two-tailed paired-samples *t*-test with N = 117, α = 0.05, and statistical power of 0.95 yielded a minimum detectable standardized within-participant effect of Cohen’s *d*_z_ = 0.336. For the hierarchical multiple regression analyses, an additional sensitivity analysis was performed for the increase in R^2^ attributable to the two variables entered in the second block after accounting for the five variables entered in the first block. With N = 117, α = 0.05, statistical power of 0.95, two tested variables and seven total variables, the minimum detectable incremental effect size was Cohen’s f^2^ = 0.136. These sensitivity analyses characterize the magnitude of effects detectable with the achieved sample and are not presented as a retrospective substitute for a prospectively specified sample-size calculation.

The study population consisted of patients aged 60 years and older, of both sexes, with a diagnosis of severe sensorineural hearing loss in both ears and an indication for the use of individual sound amplification devices (hearing aids).

Baseline eligibility criteria were: provision of written informed consent for participation in the study; age ≥ 60 years; diagnosed severe sensorineural hearing loss in both ears, with both ears meeting the predefined audiological criteria (≥70 dB at a minimum of two frequencies within the speech range: 1000, 2000, 3000, and 4000 Hz); no previous use of an individual hearing aid before completion of the baseline questionnaire; and a general health condition that allowed attendance at study visits and completion of the questionnaires.

For inclusion in the final complete-case analytical sample, participants were additionally required to complete the follow-up assessment after 3–6 months, to report regular hearing-aid use of more than six hours per day during this period, and to attend the scheduled follow-up visits for hearing-aid fine-tuning and counseling.

Exclusion criteria were: refusal to participate in the study; presence of conductive hearing loss; presence of psychiatric or cognitive disorders; and previous use of an individual hearing aid.

### 2.3. Audiological Assessment

Following the collection of medical history data and a clinical otorhinolaryngological examination, hearing assessment was performed using a MADSEN Itera II diagnostic audiometer (GN Otometrics A/S, Taastrup, Denmark) in a sound-treated room. Pure-tone audiometry was performed across the standard frequency range (125–8000 Hz), using both air and bone-conduction testing. This procedure was used to determine hearing thresholds and to establish the type and degree of hearing loss.

### 2.4. Study Instruments

Participants were assessed using Serbian-language versions of the Hearing Handicap Inventory for the Elderly (HHIE) [19], the International Outcome Inventory for Hearing Aids (IOI-HA) [20], and the World Health Organisation Quality of Life-BREF questionnaire (WHOQOL-BREF) [21,22], as well as a questionnaire assessing satisfaction with communication with healthcare professionals [23,24]. The Serbian version of the WHOQOL-BREF has previously been evaluated for validity and reliability in an elderly population in Vojvodina [22]. For the HHIE and IOI-HA, Serbian-language translated versions were used; however, comprehensive psychometric validation of these translated versions in a comparable Serbian population is limited.

Self-perceived hearing handicap was assessed using the Hearing Handicap Inventory for the Elderly (HHIE), a widely used questionnaire developed by Ventry and Weinstein [19]. The instrument comprises 25 items grouped into two subscales assessing the emotional (13 items) and social/situational (12 items) consequences of hearing impairment. Response options were “Yes”, “Sometimes”, and “No”, scored as 4, 2, and 0 points, respectively. The total score ranges from 0 to 100, with higher scores indicating a greater self-perceived hearing handicap.

Self-reported hearing aid outcomes were assessed using the International Outcome Inventory for Hearing Aids (IOI-HA) [20]. The questionnaire consists of seven items evaluating daily hearing aid use, perceived benefit, residual activity limitations, satisfaction, residual participation restrictions, impact on others, and hearing-related quality of life. Each item is rated on a five-point Likert scale, and the total score is calculated by summing the individual item scores, yielding a possible range of 7–35. Higher scores indicate more favourable self-reported hearing aid outcomes.

Quality of life was assessed using the Serbian version of the WHOQOL-BREF [19,20], whose validity and reliability have previously been evaluated in an elderly population in Vojvodina [22]. The WHOQOL-BREF consists of 26 items rated on a five-point Likert scale and assesses four domains of quality of life: physical health, psychological health, social relationships and environment. Domain scores were transformed to a 0–100 scale according to the WHOQOL-BREF scoring guidelines, with higher scores indicating better quality of life.

Satisfaction with communication with healthcare professionals was assessed using 12 patient-reported items selected from the information-related items of the patient questionnaire used by Nørgaard et al. [23], which was based on the interpersonal skills assessment described by Schnabl et al. [24]. The selected items addressed six corresponding aspects of communication with physicians and nurses/nursing assistants: preparedness for the consultation, use of understandable language, opportunity for the patient to explain the health problem, explanation of examinations and treatments, explanation of further plans, and satisfaction with the information received. Each item was rated on a five-point Likert scale ranging from 1 (strongly disagree) to 5 (strongly agree). The 12 item scores were summed to obtain a total communication satisfaction score ranging from 12 to 60, with higher scores indicating greater satisfaction with communication and information provided by healthcare professionals. The 12 items were analysed as a single composite communication satisfaction measure in the present study. This specific 12-item composite has not been formally validated as a Serbian-language patient-reported measure of healthcare communication; therefore, its internal consistency was evaluated in the present sample. Cronbach’s alpha was interpreted as an indicator of internal consistency and not as evidence of unidimensionality or construct validity.

At baseline and follow-up, communication satisfaction was assessed under standardised conditions. The same healthcare professionals were involved at both assessments, the same type of consultation was used, the assessments were conducted in the same clinical setting, and the same type of communication content was addressed. Baseline communication satisfaction was assessed before any hearing-aid counseling was provided, whereas the post-amplification assessment was conducted during a follow-up healthcare consultation rather than retrospectively.

All questionnaires were administered in Serbian in written form and were self-completed by the participants. The questionnaires were not administered orally by the research staff. The same mode of administration was used at the study assessments.

### 2.5. Hearing Amplification and Follow-Up

Following the initial assessment, the patients underwent hearing amplification.

Hearing amplification was performed using behind-the-ear (BTE) hearing aids, specifically the Unitron T Stride P 600 (Unitron Hearing, Kitchener, ON, Canada) and Bernafon Entra A1 B105 (SBO Hearing A/S, Smørum, Denmark) models. Hearing aids were prescribed through the standard OPP prescription form, and each participant received one hearing aid reimbursed by the Republic Fund of Health Insurance of Serbia.

Hearing-aid fitting was performed unilaterally, while subsequent adjustments were performed individually based on each participant’s audiogram. Hearing-aid gain was individually adjusted according to each participant’s audiometric thresholds rather than prescribed as a uniform gain value for all participants.

After a period of effective hearing aid use, defined as daily use for more than six hours per day over a period of 3–6 months, during which patients attended regular follow-up visits for fine-tuning of their hearing aids, a follow-up assessment was conducted using the same relevant instruments. This approach enabled the assessment of changes in self-perceived hearing handicap, quality of life following hearing amplification, and satisfaction with communication with healthcare professionals.

The audiological assessment protocol, hearing-aid models, fitting and verification procedures, counseling procedures, and follow-up schedule were standardized across all three participating centers.

### 2.6. Statistical Analysis

Statistical analyses were performed using IBM SPSS Statistics, version 21.0 (IBM Corp., Armonk, NY, USA). Normally distributed continuous variables are presented as mean ± standard deviation (SD), non-normally distributed variables as median and interquartile range (IQR), and categorical variables as frequencies and percentages.

Within-participant changes were calculated as post-amplification minus pre-amplification scores. The choice of paired test was based on the distribution of within-participant difference scores, assessed using the Shapiro–Wilk test and graphical inspection. Approximately normally distributed differences were analysed using paired-samples *t*-tests, whereas non-normally distributed differences were analysed using Wilcoxon signed-rank tests after examination of their shape and approximate symmetry. All pre–post analyses included 117 complete pairs. Changes are reported as mean changes with 95% confidence intervals (CIs) and Cohen’s d_z_ for paired *t*-tests, and as median changes with 95% bootstrap CIs based on 5000 resamples and effect size r for Wilcoxon tests. Holm’s sequential procedure was applied across the eight inferential pre–post comparisons (three HHIE outcomes, four WHOQOL-BREF domains, and total communication satisfaction). The 12 individual communication items were analysed descriptively.

Internal consistency was assessed using Cronbach’s alpha, interpreted as an indicator of internal consistency rather than construct validity. Item-level missingness was examined for the HHIE, WHOQOL-BREF, and IOI-HA, and floor and ceiling effects were calculated as the proportions obtaining the minimum and maximum possible scores. Associations between hearing-aid outcomes, communication satisfaction, and quality-of-life outcomes were examined using Spearman’s rank correlation coefficient (ρ).

Hierarchical multiple linear regression analyses were performed separately for each post-amplification WHOQOL-BREF domain. Model 1 included the corresponding baseline domain score, sex, age, chronic disease/comorbidity, and baseline total HHIE score; Model 2 additionally included the total IOI-HA score and post-amplification communication satisfaction score. These covariates were selected to account for baseline quality of life and relevant demographic, health-related, and hearing-related characteristics and were retained irrespective of statistical significance. No WHOQOL-BREF domain had been prespecified as the primary outcome. Holm adjustment was therefore applied separately across the four domain-specific tests of the IOI-HA coefficient and the four tests of the communication satisfaction coefficient, while correlation analyses were considered exploratory.

Regression assumptions and diagnostics were evaluated using residual-versus-predicted-value plots for linearity and homoscedasticity, examination of standardized residuals, variance inflation factors (VIFs) for multicollinearity, and Cook’s distance for influential observations. As a sensitivity analysis, final regression models were additionally evaluated using nonparametric bootstrapping with bias-corrected and accelerated (BCa) 95% CIs based on 2000 requested bootstrap samples to assess the robustness of conclusions for IOI-HA and communication satisfaction.

Primary analyses used a complete-case approach and included the 117 participants who completed follow-up and met the predefined analytical criteria. All variables required for the pre–post and multivariable analyses were available for these participants, with no additional loss due to missing data. Missing responses occurred only for selected sociodemographic characteristics used descriptively, for which percentages were calculated from available responses. All tests were two-sided, with *p* < 0.05 considered statistically significant.

## 3. Results

### 3.1. Participant Flow

**Figure 1:**
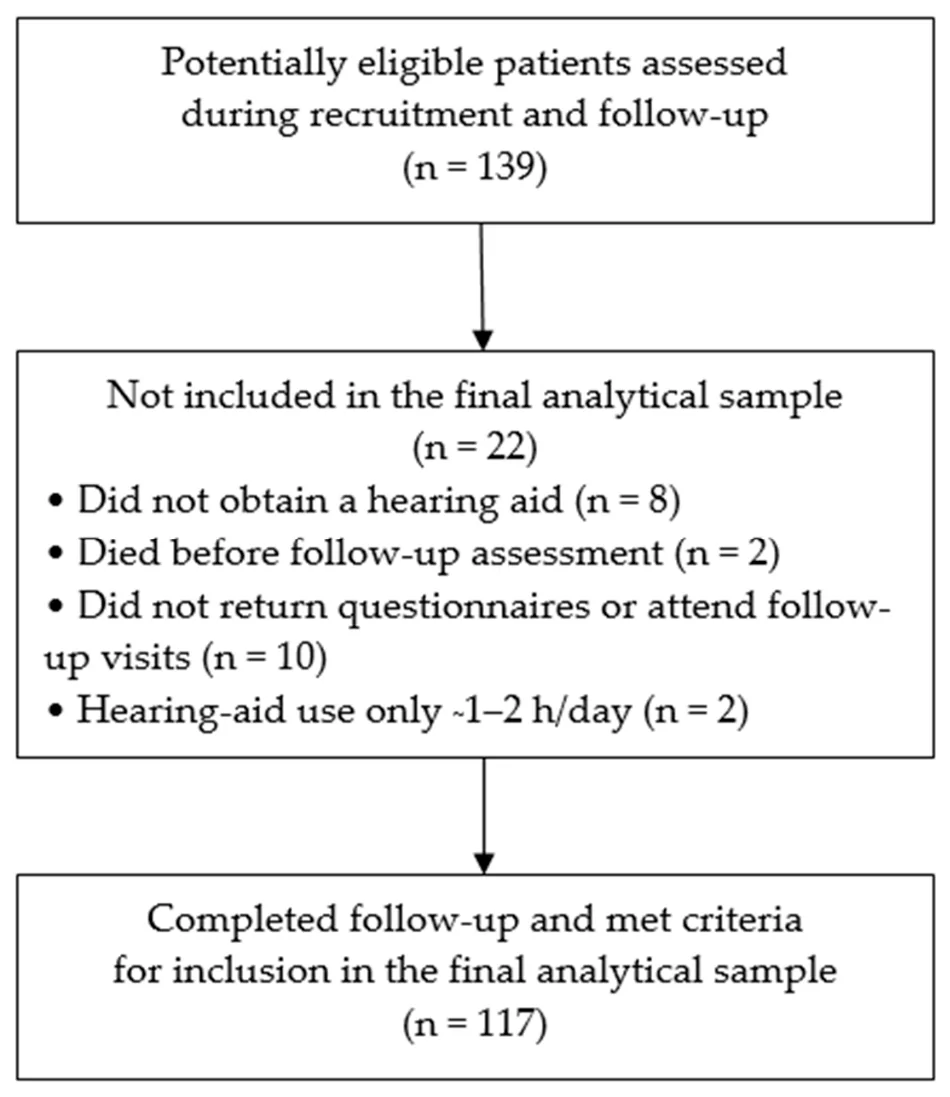
Participant flow through recruitment, hearing amplification, follow-up, and final analysis.

During the recruitment and follow-up process, 139 potentially eligible patients were assessed. Of these, 117 completed the follow-up assessment and met the predefined criteria for inclusion in the final analytical sample. The reasons for non-inclusion of the remaining 22 patients are presented in Figure 1.

### 3.2. Participant Characteristics

The sociodemographic and health-related characteristics of the study participants are presented in Table 1. A total of 117 participants were included in the study, of whom 62 (53.0%) were male and 55 (47.0%) were female. The mean age of the participants was 77.53 ± 6.46 years, ranging from 61 to 92 years.

Regarding marital status, the majority of participants were married (68; 58.1%), while 45 (38.5%) were widowed. Most participants had completed secondary school (53; 45.3%) or primary school (48; 41.0%). Among participants who provided information on monthly household income, the largest proportion reported an income below 40,000 RSD (50; 44.2%), followed by an income between 40,000 and 80,000 RSD (46; 40.7%). Most participants lived in two-person households (51; 45.1%), and more than half lived with a spouse or cohabiting partner (64; 54.7%). 80 (68.1%) participants reported a chronic disease or disability in a household member, while 91 (77.8%) participants reported having at least one chronic non-communicable disease.

### 3.3. Hearing Handicap and Quality of Life Before Hearing Amplification

Baseline hearing handicap and quality of life scores are presented in Table 2. The mean total HHIE score indicated a significant self-perceived hearing handicap at baseline. The HHIE demonstrated high internal consistency, with Cronbach’s α coefficients ranging from 0.846 to 0.925. Among the WHOQOL-BREF domains, the physical health domain had the lowest baseline score, whereas the environment domain had the highest. Cronbach’s α coefficients for the WHOQOL-BREF domains ranged from 0.574 to 0.851. The social relationships domain showed comparatively low internal consistency (Cronbach’s α = 0.574); therefore, findings involving this domain should be interpreted with caution.

### 3.4. Missing Data and Floor and Ceiling Effects

Item-level missingness across the HHIE, WHOQOL-BREF, and IOI-HA ranged from 0.0% to 23.9%. With the exception of WHOQOL-BREF item 21, item-level missingness did not exceed 6.0%. WHOQOL-BREF item 21, assessing satisfaction with sexual life, was unanswered by 22 participants (18.8%) at baseline and 28 participants (23.9%) after hearing amplification. Across the HHIE total score, WHOQOL-BREF domains, and IOI-HA total score, floor effects ranged from 0.0% to 2.6%, while ceiling effects ranged from 0.0% to 3.4%.

### 3.5. Changes in Hearing Handicap Following Hearing Amplification

Changes in self-perceived hearing handicap following hearing amplification are presented in Table 3. Statistically significant reductions were observed in the total HHIE score and in both the emotional and social/situational subscale scores (all *p* < 0.001). The mean within-participant changes were −24.63 points (95% CI: −29.24 to −20.02) for the total HHIE score and −12.15 points (95% CI: −14.85 to −9.46) for the emotional subscale, while the median within-participant change in the social/situational subscale was −10.00 points (95% bootstrap CI: −14.00 to −8.00). All three changes remained statistically significant after Holm adjustment for multiple comparisons. Effect sizes were large across the HHIE measures (d_z_ = 0.98 for the total score, d_z_ = 0.83 for the emotional subscale, and r = 0.79 for the social/situational subscale).

### 3.6. Changes in Quality of Life Following Hearing Amplification

Changes in quality of life following hearing amplification are presented in Table 4. Statistically significant improvements were observed across all WHOQOL-BREF domains. The mean increase was 5.11 points for physical health (95% CI: 1.29 to 8.92; *p* = 0.009), 9.82 points for psychological health (95% CI: 6.15 to 13.49; *p* < 0.001), 6.91 points for social relationships (95% CI: 3.47 to 10.35; *p* < 0.001), and 5.58 points for the environment domain (95% CI: 2.13 to 9.03; *p* = 0.002). All four improvements remained statistically significant after Holm adjustment for multiple comparisons. The standardized effect sizes were modest to moderate, with the largest effect observed for psychological health (d_z_ = 0.49), followed by social relationships (d_z_ = 0.37), environment (d_z_ = 0.30), and physical health (d_z_ = 0.25).

### 3.7. Self-Reported Outcomes of Hearing Aid Use

Self-reported outcomes of hearing aid use were assessed using the International Outcome Inventory for Hearing Aids (IOI-HA). The median total IOI-HA score was 27.0 (IQR = 7.5), with scores ranging from 12 to 35. The IOI-HA demonstrated high internal consistency, with a Cronbach’s α coefficient of 0.885.

### 3.8. Satisfaction with Communication with Healthcare Professionals

**Figure 2:**
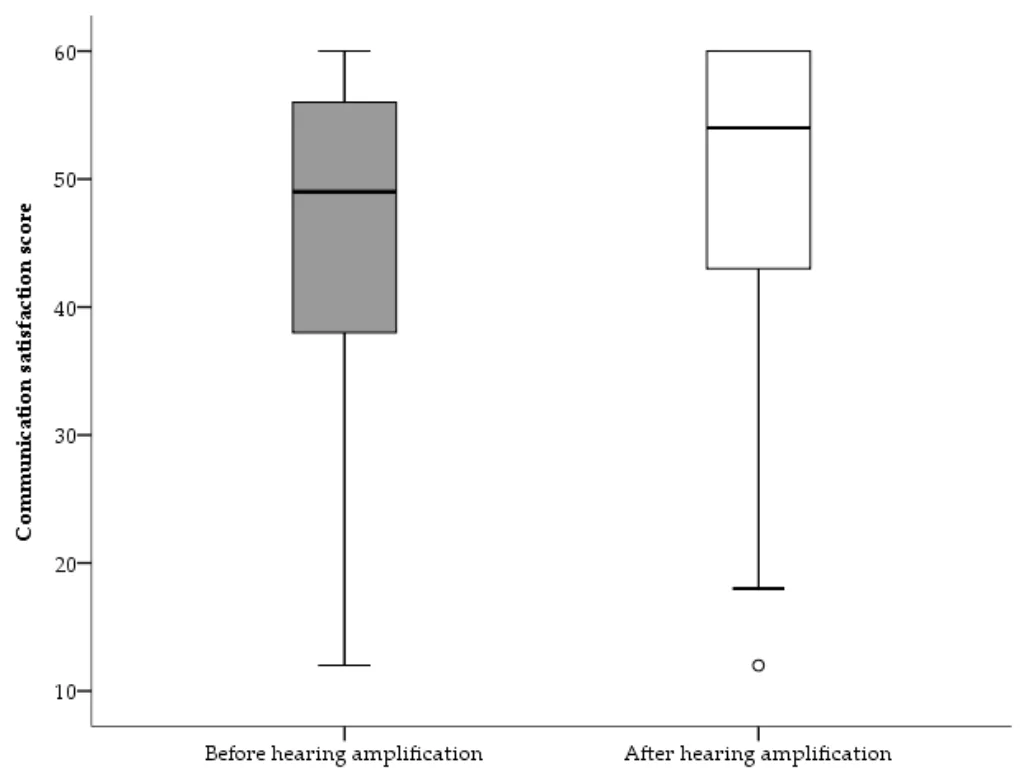
Distribution of total communication satisfaction scores before and after hearing amplification.

Satisfaction with communication with healthcare professionals before and after hearing amplification is presented in Table 5, while the distribution of total communication satisfaction scores at the two assessment points is illustrated in Figure 2. Mean scores were higher after hearing amplification across all individual communication items. Among 117 complete pairs, the median total communication satisfaction score increased from 49.0 (IQR = 18.5) before amplification to 54.0 (IQR = 17.5) after amplification. The median within-participant change was 2.00 points (95% bootstrap CI: 0.00 to 3.00), and the change was statistically significant (Z = −3.590, *p* < 0.001; Holm-adjusted *p* < 0.001), with an effect size of r = 0.37. The 12-item communication satisfaction composite measure demonstrated high internal consistency both before and after hearing amplification, with Cronbach’s α coefficients of 0.959 and 0.970, respectively. A ceiling effect was observed for the total communication satisfaction score, with 20 participants (17.1%) achieving the maximum possible score of 60 at baseline and 39 participants (33.3%) achieving the maximum score after hearing amplification.

### 3.9. Association of Hearing Aid Outcomes with Quality of Life

The associations between self-reported hearing aid outcomes and quality of life following hearing amplification are presented in Table 6. Higher total IOI-HA scores were significantly associated with higher post-amplification scores across all WHOQOL-BREF domains. The largest correlation coefficient was observed for the environment domain (ρ = 0.323), followed by social relationships (ρ = 0.304), psychological health (ρ = 0.257) and physical health (ρ = 0.222).

Higher total IOI-HA scores were also significantly associated with greater improvements in the physical health (ρ = 0.236), psychological health (ρ = 0.217) and social relationships (ρ = 0.275) domains. No statistically significant association was observed between the total IOI-HA score and change in the environment domain (ρ = 0.170).

### 3.10. Association of Post-Amplification Satisfaction with Communication with Quality of Life

The associations between satisfaction with communication with healthcare professionals after hearing amplification and quality of life are presented in Table 7. Higher post-amplification communication satisfaction scores were significantly associated with higher post-amplification quality of life across all WHOQOL-BREF domains. The largest correlation coefficient was observed for social relationships (ρ = 0.399), followed by environment (ρ = 0.344), physical health (ρ = 0.274), and psychological health (ρ = 0.247).

### 3.11. Association Between Changes in Communication Satisfaction and Changes in Quality of Life

The associations between changes in satisfaction with communication with healthcare professionals and changes in quality of life following hearing amplification are presented in Table 8. Changes in communication satisfaction were not significantly associated with changes in any of the WHOQOL-BREF domains (*p* > 0.05).

### 3.12. Hierarchical Linear Regression Analysis of Factors Associated with Physical Health After Hearing Amplification

Hierarchical linear regression analysis was performed to identify factors independently associated with physical health after hearing amplification. In Model 1, baseline physical health, gender, age, chronic disease/comorbidity, and baseline hearing handicap explained 28.4% of the variance in post-amplification physical health (R^2^ = 0.284, adjusted R^2^ = 0.252; *p* < 0.001). Baseline physical health and gender were significantly associated with post-amplification physical health in this model.

After the addition of the IOI-HA total score and post-amplification communication satisfaction score in Model 2, the proportion of explained variance increased to 40.3% (R^2^ = 0.403, adjusted R^2^ = 0.364). The addition of these variables explained an additional 11.8% of the variance (ΔR^2^ = 0.118, F change = 10.774, *p* < 0.001). In the final model, a higher IOI-HA total score was independently associated with better physical health (β = 0.310, *p* < 0.001). In contrast, post-amplification communication satisfaction was not significantly associated with physical health (β = 0.092, *p* = 0.259). Baseline physical health, gender, age, and chronic disease/comorbidity were also significantly associated with post-amplification physical health in the final model, whereas baseline hearing handicap was not (Table 9).

### 3.13. Hierarchical Linear Regression Analysis of Factors Associated with Psychological Health After Hearing Amplification

Hierarchical linear regression analysis was performed to identify factors independently associated with psychological health after hearing amplification. In Model 1, baseline psychological health, gender, age, chronic disease/comorbidity and baseline hearing handicap explained 30.7% of the variance in post-amplification psychological health (R^2^ = 0.307, adjusted R^2^ = 0.276; *p* < 0.001). Baseline psychological health, gender, age and chronic disease/comorbidity were significantly associated with post-amplification psychological health in this model, whereas baseline hearing handicap was not.

After the addition of the IOI-HA total score and post-amplification communication satisfaction score in Model 2, the proportion of explained variance increased to 44.7% (R^2^ = 0.447, adjusted R^2^ = 0.411), representing an additional 13.9% of the explained variance (ΔR^2^ = 0.139, F change = 13.733, *p* < 0.001). In the final model, a higher IOI-HA total score was independently associated with better psychological health (β = 0.331, *p* < 0.001). In contrast, communication satisfaction was not significantly associated with psychological health (β = 0.111, *p* = 0.156). Baseline psychological health, gender, age, chronic disease/comorbidity and baseline hearing handicap were also significantly associated with post-amplification psychological health in the final model (Table 10).

### 3.14. Hierarchical Linear Regression Analysis of Factors Associated with Social Relationships After Hearing Amplification

Hierarchical linear regression analysis was performed to identify factors independently associated with social relationships after hearing amplification. In Model 1, baseline social relationships, gender, age, chronic disease/comorbidity, and baseline hearing handicap explained 24.0% of the variance in post-amplification social relationships (R^2^ = 0.240, adjusted R^2^ = 0.206; *p* < 0.001). Baseline social relationships, gender and age were significantly associated with post-amplification social relationships in this model, whereas chronic disease/comorbidity and baseline hearing handicap were not.

After the addition of the IOI-HA total score and post-amplification communication satisfaction score in Model 2, the proportion of explained variance increased to 40.3% (R^2^ = 0.403, adjusted R^2^ = 0.364), representing a significant additional 16.2% of explained variance (ΔR^2^ = 0.162, F change = 14.783, *p* < 0.001). In the final model, a higher IOI-HA total score (β = 0.288, *p* < 0.001) and greater post-amplification communication satisfaction (β = 0.221, *p* = 0.008) were independently associated with higher scores in the social relationships domain. Baseline social relationships and age were also significantly associated with post-amplification social relationships in the final model, whereas gender, chronic disease/comorbidity and baseline hearing handicap were not (Table 11).

### 3.15. Hierarchical Linear Regression Analysis of Factors Associated with Environment Domain Scores After Hearing Amplification

Hierarchical linear regression analysis was performed to identify factors independently associated with environment domain scores after hearing amplification. In Model 1, baseline environment score, gender, age, chronic disease/comorbidity, and baseline hearing handicap explained 23.3% of the variance in post-amplification scores for the environment domain (R^2^ = 0.233, adjusted R^2^ = 0.199; *p* < 0.001). Baseline environment score and age were significantly associated with post-amplification scores for the environment domain in this model, whereas gender, chronic disease/comorbidity, and baseline hearing handicap were not.

After the addition of the IOI-HA total score and post-amplification communication satisfaction score in Model 2, the proportion of explained variance increased to 35.2% (R^2^ = 0.352, adjusted R^2^ = 0.310), representing a significant additional 11.9% of explained variance (ΔR^2^ = 0.119, F change = 9.971, *p* < 0.001). In the final model, a higher IOI-HA total score was independently associated with higher environment domain scores (β = 0.290, *p* = 0.001). In contrast, post-amplification communication satisfaction was not significantly associated with environment domain scores (β = 0.134, *p* = 0.120). Baseline environment score and age were also significantly associated with post-amplification scores for the environment domain in the final model, whereas gender, chronic disease/comorbidity, and baseline hearing handicap were not (Table 12).

After Holm adjustment across the four domain-specific regression models, the associations between the IOI-HA score and all four WHOQOL-BREF domains remained statistically significant (all adjusted *p* < 0.001). For post-amplification communication satisfaction, only the association with the social relationships domain was statistically significant in the unadjusted analysis (*p* = 0.008), and this association remained statistically significant after Holm adjustment (adjusted *p* = 0.032). Communication satisfaction was not significantly associated with the physical health, psychological health, or environment domains.

### 3.16. Regression Diagnostics

Regression diagnostics were performed for all four hierarchical linear regression models. Visual inspection of residual-versus-predicted-value plots showed no clear evidence of substantial non-linearity or heteroscedasticity. Histograms and normal P–P plots of standardised residuals did not indicate substantial departures from normality. Multicollinearity was low across all models (all VIF values < 1.3). No potentially influential observations were identified based on Cook’s distance, with maximum values of 0.071, 0.106, 0.068, and 0.094 for the physical health, psychological health, social relationships, and environment domain models, respectively. No standardised residuals exceeded ±3 in any of the models. Bootstrap sensitivity analyses were consistent with the conventional regression results. The IOI-HA coefficient remained positive, with BCa 95% confidence intervals excluding zero, for physical health (B = 1.031, 95% BCa CI: 0.446 to 1.503), psychological health (B = 1.007, 95% BCa CI: 0.445 to 1.554), social relationships (B = 0.882, 95% BCa CI: 0.368 to 1.391), and environment (B = 0.873, 95% BCa CI: 0.395 to 1.381). For post-amplification communication satisfaction, the BCa 95% confidence interval excluded zero only for the social relationships domain (B = 0.339, 95% BCa CI: 0.082 to 0.572), whereas the intervals included zero for physical health (B = 0.152, 95% BCa CI: −0.068 to 0.394), psychological health (B = 0.168, 95% BCa CI: −0.089 to 0.410), and environment (B = 0.201, 95% BCa CI: −0.048 to 0.498). Thus, the bootstrap sensitivity analyses did not materially alter the conclusions of the conventional regression analyses.

## 4. Discussion

Considering the aim of assessing changes in self-perceived hearing handicap and quality of life among older adults following hearing amplification, as well as the associations of hearing aid outcomes and satisfaction with communication with healthcare professionals with quality of life, the results of this study revealed several important findings. Following hearing amplification, lower levels of self-perceived hearing handicap and higher scores across all four quality-of-life domains were observed, along with favourable hearing aid outcomes and greater satisfaction with communication with healthcare professionals. One of the key findings was the independent association between the IOI-HA score and all four quality-of-life domains. In contrast, satisfaction with communication with healthcare professionals was independently associated only with the social relationships domain. These findings underscore the importance of considering subjective hearing aid outcomes in a comprehensive assessment of hearing rehabilitation, while also suggesting that the association between satisfaction with communication and quality of life may be specific to its social dimension.

These findings should be interpreted in the context of the uncontrolled pre–post study design. In the absence of a control group, the observed changes cannot be attributed specifically to hearing amplification, as alternative explanations including regression to the mean, maturation, repeated-measurement effects, Hawthorne effects, concurrent changes in health status, and placebo or expectation effects cannot be excluded. Moreover, hearing amplification was delivered as part of a broader rehabilitation process that included regular follow-up visits, repeated counseling, and hearing-aid fine-tuning. Therefore, the observed improvements may reflect the combined effects of the overall hearing rehabilitation program rather than amplification alone.

In the present study, hearing amplification was followed by a significant reduction in self-perceived hearing handicap, as reflected in the total HHIE score as well as in the emotional and social/situational domains. The observed reductions in HHIE scores were accompanied by large standardized effect sizes. However, standardized effect sizes reflect the magnitude of the observed pre–post differences relative to their variability and should not, by themselves, be interpreted as evidence of clinical importance. Therefore, the absolute within-participant changes and their confidence intervals were also considered when interpreting these findings. An established minimally important difference that could be directly applied to the HHIE scores in the present population and study context was not identified; therefore, the observed changes were not classified as clinically important on the basis of a specific threshold. These findings are consistent with previous studies reporting lower HHIE and HHIE-S scores following hearing rehabilitation [25,26,27,28,29]. Similar findings have also been reported in randomised trials, including the ACHIEVE study, in which improvements in self-perceived communication function were evident as early as six months and were sustained throughout the three-year follow-up period [26,27]. The present findings are also consistent with a systematic review of randomised trials demonstrating favourable effects of hearing aid use on hearing-specific quality of life and participation in everyday activities [30].

A large standardized effect was observed for the social/situational domain (r = 0.79). One possible explanation for this finding may lie in the importance of hearing for everyday communication and social functioning, given that hearing impairment in older adults is associated with communication difficulties and restrictions in social participation, as well as loneliness and social isolation [31,32]. Previous research also suggests that hearing aid use may be accompanied by fewer restrictions in social participation and greater social activity [33]. The observed change in the social/situational domain may therefore reflect a more favourable perception of one’s own functioning in everyday social and communication situations following hearing amplification, highlighting the importance of assessing functional and psychosocial outcomes in older adults with hearing impairment.

The potential relevance of hearing rehabilitation to cognitive health should also be considered in this context. Recent longitudinal evidence has shown that hearing loss is associated with an increased risk of dementia, while lower risk estimates have been observed among individuals with hearing loss who use hearing aids compared with those who do not [34]. Recent evidence has also highlighted the potential relevance of social pathways, showing that hearing impairment accompanied by social isolation and limited conversational interaction is associated with an increased risk of dementia [35]. These findings provide a broader context for the marked reduction in the social/situational component of self-perceived hearing handicap observed in the present study. However, cognitive function, incident dementia, and changes in social participation were not directly assessed; therefore, our findings cannot establish whether hearing amplification reduced dementia risk or whether the observed improvement in hearing-related social functioning translated into cognitive benefits.

Following hearing amplification, statistically significant improvements were observed across all four quality-of-life domains. The standardized effect sizes were modest to moderate, while the absolute changes varied across domains. The greatest improvement was observed in the psychological health domain, while smaller changes were observed in the social relationships, environment, and physical health domains. These findings should therefore be interpreted cautiously and should not be taken as evidence of large changes in general quality of life. An established minimally important difference that could be directly applied to the WHOQOL-BREF domain scores in the present population and study context was not identified; therefore, clinical importance was not inferred from statistical significance or standardized effect sizes alone. These findings are consistent with previous studies reporting improvements in quality of life following hearing aid fitting and use, including studies in which the WHOQOL-BREF was administered [36,37]. Previous findings also suggest that changes across individual quality-of-life domains may not be equally pronounced, highlighting the importance of considering each domain separately when evaluating the outcomes of hearing rehabilitation [38].

The relatively greater improvement in the psychological health domain observed in our study may be interpreted within the broader context of the psychosocial aspects of hearing impairment in older adults. Previous research has demonstrated associations between hearing impairment and hearing aid outcomes, as well as various aspects of psychological well-being and quality of life [39,40], while a systematic review of the literature has highlighted the association of hearing impairment with loneliness and social isolation [32]. In contrast, the physical health domain showed a comparatively smaller standardized effect. This finding should be interpreted within a broader geriatric context. Quality of life in older adults is multidimensional and may be influenced not only by hearing status but also by multimorbidity, frailty, cognitive functioning, depressive symptoms, mobility and functional limitations, social networks, and socioeconomic circumstances. In the present sample, the mean age was 77.5 years and 77.8% of participants had at least one chronic non-communicable disease. However, the burden and severity of multimorbidity, frailty, functional impairment, cognitive status, and depressive symptoms were not comprehensively characterized. The modest improvement observed in the physical health domain should therefore be interpreted cautiously, as this dimension of quality of life is likely to reflect multiple aspects of health and functioning that may not be substantially modified by hearing rehabilitation alone. Similarly, the domain-specific associations observed in this study should be interpreted within the broader health and psychosocial context of older adults rather than as evidence that hearing amplification independently modifies general health-related quality of life. At the same time, the observed changes in the psychological and social domains underscore the importance of considering the psychosocial dimensions of quality of life among older adults with hearing impairment.

An additional important finding of this study concerns the association between hearing aid outcomes and quality of life following hearing amplification. A higher total IOI-HA score was associated with higher scores across all four WHOQOL-BREF domains after amplification, as well as with changes in the physical, psychological and social relationships domains. These findings are consistent with previous studies demonstrating positive associations between hearing aid outcomes, as assessed using the IOI-HA and various aspects of quality of life [41,42].

The results of the multivariable analyses in our study further extend these observations. After accounting for baseline quality of life in the corresponding domain, sex, age, the presence of chronic disease/comorbidity, baseline hearing handicap and satisfaction with communication with healthcare professionals, the IOI-HA remained independently associated with the physical health, psychological health, social relationships and environment domains. However, because the IOI-HA score and post-amplification quality-of-life outcomes were assessed within the same post-amplification period, these findings represent adjusted cross-sectional associations among post-amplification variables and should not be interpreted as evidence that hearing aid outcomes influenced subsequent quality of life. The temporal direction of these associations cannot be established, and reverse causation is possible, whereby participants with better quality of life may also report more favourable hearing aid outcomes. These findings also highlight the relevance of distinguishing between simply receiving a hearing aid and the subjectively perceived outcomes of its use. The IOI-HA encompasses multiple dimensions of the user experience, including daily use, perceived benefit, satisfaction, residual activity limitations and participation restrictions, impact on others, and perceived quality of life [43,44]. This conceptual breadth should also be considered when interpreting the observed associations with WHOQOL-BREF. In particular, the IOI-HA includes items addressing participation restrictions and hearing-related quality of life, which conceptually overlap with aspects of quality of life assessed by the WHOQOL-BREF. Furthermore, both instruments are patient-reported measures assessed within the same post-amplification period. Consequently, part of the observed associations may reflect construct overlap and shared method variance rather than associations between entirely distinct constructs. The associations involving the IOI-HA total score should therefore be interpreted cautiously and should not be regarded as evidence of an independent effect of hearing-aid outcomes on general quality of life. Previous studies have also shown that hearing aid outcomes vary among users and are associated with various user characteristics, patterns of hearing aid use and satisfaction with hearing aid functioning in everyday situations [45,46]. Taken together, these findings support the relevance of assessing subjective hearing-aid outcomes as one component of a broader, multidimensional evaluation of hearing rehabilitation, while recognizing that such patient-reported outcomes are not conceptually independent of quality of life.

The present study focused primarily on hearing aid-related outcomes and did not separately evaluate other components of hearing rehabilitation, such as social support, auditory training, or the use of other hearing assistive devices. Therefore, the findings should not be interpreted as reflecting all components of a comprehensive hearing rehabilitation program. Future studies should consider these additional components to provide a more comprehensive assessment of hearing rehabilitation outcomes.

A distinct aspect of this study concerns satisfaction with communication with healthcare professionals, which was significantly higher following hearing amplification, with a moderate effect size (r = 0.37). However, this observed increase should be interpreted cautiously. Although improved audibility may have contributed to greater communication satisfaction, the uncontrolled study design does not allow this explanation to be distinguished from other possible influences, including repeated contact with healthcare professionals, counseling, increased familiarity with the questionnaire, or a generally more favourable evaluation following receipt of a valued intervention. The significance of this finding should be considered in the context of previous research indicating that hearing impairment may represent an important barrier to effective communication in healthcare settings and may be associated with less favourable ratings of physician communication and satisfaction with healthcare [8,47,48]. Qualitative and observational studies have further highlighted the practical difficulties experienced by individuals with hearing impairment during interactions with healthcare professionals, including misunderstanding of information and insufficient adaptation of communication to their hearing needs [9,49]. These findings, together with evidence from systematic reviews [50,51], emphasise the importance of recognising and addressing the communication needs of older adults with hearing impairment in healthcare settings.

Of particular interest is the pattern of associations between satisfaction with communication and quality of life observed in our study. Greater satisfaction with communication following amplification was associated with higher scores across all four WHOQOL-BREF domains, with the strongest association observed for the social relationships domain. After accounting for baseline quality of life, demographic and health characteristics, baseline hearing handicap and IOI-HA outcomes, satisfaction with communication remained independently associated only with the social relationships domain. This finding may reflect the conceptual proximity between satisfaction with interpersonal communication and the social dimension of quality of life. It may be interpreted within the broader context of the importance of communication for social functioning in older adults, given that communication difficulties have been associated with smaller social networks, fewer positive social interactions, lower participation in social activities and greater loneliness [52]. However, changes in satisfaction with communication in our study were not significantly associated with changes in any quality-of-life domain. Therefore, the present findings suggest that satisfaction with communication with healthcare professionals may represent a relevant correlate of the social dimension of quality of life. Still, they do not support the assumption that changes in communication satisfaction per se constitute a mechanism underlying improvements in quality of life following hearing amplification. Because communication satisfaction and post-amplification quality of life were assessed within the same post-amplification period, the temporal ordering of this association cannot be established. Accordingly, the observed association with the social relationships domain should be interpreted as an adjusted cross-sectional relationship rather than evidence of a mediating pathway. This finding further underscores the importance of communication tailored to the needs of older adults with hearing impairment, while also highlighting the need for further research to clarify its relationship with different aspects of quality of life.

Baseline quality of life was also significantly associated with post-amplification quality of life across all four domains. In contrast, age, sex and the presence of chronic disease/comorbidity showed different patterns of association depending on the domain examined. Baseline self-perceived hearing handicap, however, was generally not independently associated with post-amplification quality of life after the other factors were included in the models, except in the psychological health domain. In contrast, the IOI-HA remained independently associated with all four quality-of-life domains. This distinction suggests that the baseline severity of subjectively perceived hearing handicap and the outcomes of hearing aid use represent different aspects of the experience of individuals with hearing impairment. Whereas the HHIE reflects the perceived consequences of hearing impairment before amplification, the IOI-HA captures outcomes related to the actual use of hearing aids in everyday life. Previous studies have likewise indicated that hearing aid outcomes are multifactorial and associated with individual user characteristics and experiences, as well as with patterns of hearing aid use and hearing aid functioning in everyday situations [45,46]. The present findings therefore suggest that the baseline severity of hearing handicap alone was not consistently associated with quality of life following amplification and that the actual, subjectively perceived outcomes of hearing aid use should also be taken into consideration.

The findings of this study have clinical and practical implications for the assessment of hearing rehabilitation in older adults. The multidimensional approach used in this study supports considering patient-reported hearing-aid outcomes, quality of life, and communication needs alongside audiological assessment. However, the present findings relate specifically to participants’ satisfaction with communication with healthcare professionals and should not be interpreted as evidence of improved objective communication quality, comprehension of medical information, shared decision-making, medication adherence, healthcare safety, treatment outcomes, or health-service equity. These outcomes were not assessed in the present study. Accordingly, the observed increase in communication satisfaction should be interpreted as a patient-reported communication outcome rather than as evidence of broader improvements in healthcare delivery or clinical outcomes. Within these limits, the findings support a patient-centred approach to hearing rehabilitation that considers users’ experiences with hearing-aid use and their communication needs within healthcare settings.

The strengths of this study include the prospective pre–post assessment of hearing amplification outcomes and the multidimensional approach encompassing self-perceived hearing handicap, different domains of quality of life, hearing aid outcomes, and satisfaction with communication with healthcare professionals. An additional strength is the reporting of effect sizes, as well as the use of hierarchical regression models, which enabled the assessment of the independent associations of IOI-HA outcomes and satisfaction with communication with post-amplification quality of life while controlling for baseline quality of life and relevant demographic, health-related, and hearing-related characteristics.

Several limitations should be considered when interpreting the findings of this study. First, the absence of a control group prevents attribution of the observed pre–post changes specifically to hearing amplification. The study used a within-participant pre-post design, in which the same participants were assessed before and after hearing amplification and each participant therefore served as his or her own reference. This design allowed changes following amplification to be evaluated within individuals and reduced the influence of stable between-person differences. Nevertheless, regression to the mean, repeated-measurement effects, changes in health status, expectation effects, and the accompanying counseling, follow-up, and hearing-aid fine-tuning may have contributed to the observed improvements. Accordingly, the paired pre-post design does not substitute for an independent control group, and the observed changes cannot be attributed exclusively to hearing amplification. To reduce overinterpretation arising from this limitation, the findings were framed as within-participant changes and associations rather than as evidence of a causal effect of hearing amplification.

In addition, the final analytical sample comprised participants who obtained hearing aids, completed follow-up, and reported regular use of more than six hours per day, potentially selecting relatively successful and adherent hearing-aid users. Baseline outcome data were unavailable for the 22 patients not included in the final analytical sample; therefore, comparisons between completers and non-completers and sensitivity analyses for missing follow-up outcomes could not be performed. The potential influence of selection and attrition bias consequently cannot be excluded. Restricting the analysis to participants who completed follow-up and met the predefined criterion for regular hearing-aid use ensured that post-amplification outcomes were assessed among participants with meaningful exposure to hearing amplification. However, this approach does not eliminate selection or attrition bias and may limit the applicability of the findings to less adherent hearing-aid users.

Participants were recruited from three specialist outpatient clinics, predominantly from a single center, rather than from a population-based sampling frame. Generalizability is therefore limited, particularly to older adults with milder hearing loss, cognitive or functional impairment, frailty, institutional residence, limited health literacy, or reduced access to audiological services. Moreover, eligibility was restricted to severe bilateral sensorineural hearing loss, and associations between individual audiometric thresholds and patient-reported outcomes were not examined. Although age was included as a covariate, the study was not designed to compare outcomes across different levels of hearing-loss severity. These restrictions primarily affect the external validity of the findings; therefore, the observed associations should not be assumed to extend to older adults with different clinical characteristics, degrees of hearing loss, or patterns of access to audiological care.

All primary outcomes were self-reported and may therefore be affected by response bias. Pre–post assessments may also have been susceptible to response-shift bias, as participants’ standards or expectations could have changed after experience with hearing aids. Measurement-related limitations include a ceiling effect for communication satisfaction, relatively low internal consistency of the WHOQOL-BREF social relationships domain (Cronbach’s α = 0.574), substantial missingness for its sexual-life item, and the absence of formal validation of the 12-item Serbian-language communication satisfaction composite. Accordingly, findings involving communication satisfaction and the social relationships domain should be interpreted cautiously. The use of standardized instruments and standardized assessment procedures provided consistent measurement across participants and assessment points; however, these procedures do not eliminate response, common-method, or response-shift bias. Accordingly, findings based on self-reported outcomes were interpreted as patient-reported changes and associations rather than as objective evidence of functional improvement.

Although the regression models adjusted for baseline quality of life and selected demographic, health-related, and hearing-related characteristics, residual confounding remains possible. Socioeconomic and living characteristics were not included in the models, while potentially relevant factors such as frailty, functional status, psychological symptoms, cognitive functioning, hearing-related comorbidities, and detailed characteristics of hearing-aid use were not comprehensively assessed. Cognitive function, in particular, was not evaluated using a standardized measure despite the exclusion of participants with known cognitive disorders. Adjustment for baseline quality of life and selected relevant demographic, health-related, and hearing-related covariates was used to reduce measured confounding. However, because unmeasured and residual confounding could not be excluded, the adjusted estimates were interpreted as associations rather than causal effects.

Potential construct overlap and shared method variance should also be considered because the IOI-HA includes participation and hearing-related quality-of-life components, while both the IOI-HA and WHOQOL-BREF are self-reported. Objective measures of hearing-aid use and post-amplification audiological performance were not available. Furthermore, the exact individual follow-up duration within the 3–6-month period was not systematically recorded in the analytical dataset and therefore could not be included in the statistical analyses. Differences in follow-up duration may have influenced the observed outcomes and associations. The IOI-HA, communication satisfaction, and WHOQOL-BREF outcomes were assessed within the same post-amplification period; therefore, their regression associations are cross-sectional and do not establish temporal direction or causality, and reverse causation cannot be excluded. Accordingly, these regression models were used to identify adjusted correlates of post-amplification quality of life rather than predictors, mediators, or causal determinants, thereby limiting interpretation to what is supported by the temporal structure of the data.

Finally, although participants were recruited from three institutions, institution-level identifiers were not retained in the analytical dataset, preventing formal evaluation or adjustment for potential center-level clustering. Because center-level clustering could not be quantified or adjusted for, this source of uncertainty remains unresolved and should be considered when interpreting the precision of the estimates. Heteroscedasticity was assessed visually rather than using a formal test or heteroscedasticity-robust standard errors; however, regression assumptions were systematically evaluated using residual diagnostics, standardized residuals, VIFs, and Cook’s distance, and bootstrap sensitivity analyses were additionally performed to evaluate the robustness of the regression findings. The consistency of the bootstrap and conventional regression results provides additional support for the robustness of the main regression conclusions, although these analyses do not address the other potential sources of bias described above.

Taken together, these methodological and analytical strategies reduce the potential impact of several limitations on interpretation but do not eliminate the inherent constraints of the uncontrolled pre-post design. The findings should therefore be interpreted primarily as evidence of within-participant changes following hearing amplification and adjusted associations among post-amplification outcomes, rather than as evidence of causal effects.

Future studies should address these limitations using controlled longitudinal designs with clear temporal ordering of exposures, potential mediators, and outcomes. Where feasible, randomized designs comparing immediate with delayed hearing amplification or usual care would provide stronger evidence regarding changes attributable to hearing rehabilitation. Future research should also incorporate objective hearing-aid datalogging and audiological measures such as speech-in-noise performance, together with standardized assessments of cognitive function, frailty, and other relevant geriatric characteristics. Future studies should also consider including the Speech, Spatial and Qualities of Hearing Scale (SSQ), in its full or validated short form, to provide a more detailed assessment of listening abilities and communication difficulties across everyday situations. The use of validated measures of healthcare communication would allow patient-reported communication satisfaction to be distinguished from objective communication quality, comprehension, and shared decision-making. Longer follow-up would also be valuable for assessing the persistence of observed changes. Mediation analyses should be considered only in studies with appropriate temporal ordering and sufficient sample size to support evaluation of potential mechanisms.

## 5. Conclusions

Hearing amplification was associated with a reduction in self-perceived hearing handicap and statistically significant improvements across all domains of quality of life among older adults with hearing loss. Improvements were also observed in satisfaction with communication with healthcare professionals following amplification. Importantly, better self-reported hearing aid outcomes were independently associated with higher post-amplification physical, psychological, social, and environmental quality of life, even after accounting for baseline quality of life and relevant demographic, health-related, and hearing-related characteristics. Post-amplification satisfaction with communication with healthcare professionals was an adjusted correlate of the social relationships domain; however, changes in communication satisfaction were not associated with changes in quality of life, and the findings do not establish communication satisfaction as a mediator or mechanism of quality-of-life improvement. This finding should also be interpreted cautiously given the lower internal consistency and greater item-level missingness observed within the social relationships domain. Taken together, these findings highlight the potential relevance of a comprehensive, patient-centred approach to hearing rehabilitation that considers patient-reported hearing-aid outcomes, quality of life, and communication experiences within healthcare settings. However, given the uncontrolled pre-post design and the potential for selection, measurement, and residual confounding biases, the observed improvements should not be interpreted as effects attributable solely to hearing amplification. Rather, they represent within-participant changes observed following hearing amplification within the broader hearing-rehabilitation process.

## Figures and Tables

**Table 1:** Sociodemographic and health-related characteristics of the study participants (N = 117).

Characteristics	n (%)/Mean ± SD
**Age (years)**	77.53 ± 6.46
**Gender**	
Male	62 (53.0)
Female	55 (47.0)
**Marital status**	
Married	68 (58.1)
Cohabiting	1 (0.9)
Divorced	3 (2.6)
Widowed	45 (38.5)
**Educational level**	
No formal education	4 (3.4)
Primary school	48 (41.0)
Secondary school	53 (45.3)
College/University	11 (9.4)
Master’s/Doctoral degree	1 (0.9)
**Monthly household income (RSD)**	
<40,000	50 (44.2)
40,000–80,000	46 (40.7)
80,001–120,000	13 (11.5)
>120,000	2 (1.8)
Prefer not to answer	2 (1.8)
**Household size**	
One person	24 (21.1)
Two persons	51 (45.1)
≥3 persons	38 (33.6)
**Living arrangement**	
Alone	24 (20.5)
With spouse/cohabiting partner	64 (54.7)
With children	25 (21.4)
With other relatives	3 (2.6)
Other	1 (0.9)
**Chronic disease or disability in the household**	
Yes	80 (68.1)
No	37 (31.9)
**Presence of chronic non-communicable disease**	
Yes	91 (77.8)
No	26 (22.2)

Note: Continuous variables are presented as mean ± standard deviation (SD), and categorical variables as number (percentage). Percentages were calculated using available responses. Missing responses occurred for monthly household income (4/117, 3.4%) and household size (4/117, 3.4%).

**Table 2:** Hearing handicap, quality of life and internal consistency of the instruments before hearing amplification.

Measure	Baseline Score	Cronbach’s α
**HHIE**		
Emotional subscale	32.00 (22.00)	0.886
Social/situational subscale	32.00 (20.00)	0.846
Total score	62.56 ± 24.42	0.925
**WHOQOL-BREF**		
Physical health	51.10 ± 18.58	0.851
Psychological health	57.44 ± 18.35	0.843
Social relationships	58.33 (25.00)	0.574
Environment	59.54 ± 17.55	0.841

Note: Data are presented as mean ± standard deviation (SD) for normally distributed variables and as median (interquartile range, IQR) for non-normally distributed variables. WHOQOL-BREF domain scores were transformed to a 0–100 scale, with higher scores indicating better quality of life. Higher HHIE scores indicate a greater self-perceived hearing handicap. Cronbach’s α coefficients indicate the internal consistency of the respective scales and subscales at baseline. HHIE, Hearing Handicap Inventory for the Elderly; WHOQOL-BREF, World Health Organisation Quality of Life-BREF.

**Table 3:** Changes in hearing handicap following hearing amplification.

HHIE Measure	Before Amplification	After Amplification	Change (95% CI)	Test Statistic	*p*-Value	Holm-Adjusted *p*-Value	Effect Size
Emotional subscale	31.49 ± 13.67	19.33 ± 12.61	−12.15 (−14.85 to −9.46)	t = −8.933	<0.001	<0.001	d_z_ = 0.83
Social/situational subscale	32.00 (20.00)	18.00 (16.00)	−10.00 (−14.00 to −8.00) *	Z = −8.327	<0.001	<0.001	r = 0.79
Total score	62.56 ± 24.42	37.93 ± 21.83	−24.63 (−29.24 to −20.02)	t = −10.580	<0.001	<0.001	d_z_ = 0.98

Note: Values are presented as mean ± SD for outcomes analysed using paired-samples *t*-tests and median (IQR) for outcomes analysed using the Wilcoxon signed-rank test. All analyses included 117 complete pairs. Change was calculated as post-amplification minus pre-amplification score; negative values indicate a reduction in hearing handicap. * For the social/situational subscale, the 95% confidence interval refers to the bootstrap confidence interval for the median within-participant change based on 5000 bootstrap samples. HHIE, Hearing Handicap Inventory for the Elderly; CI, confidence interval.

**Table 4:** Changes in quality of life following hearing amplification.

WHOQOL-BREF Domain	Before Amplification, Mean ± SD	After Amplification, Mean ± SD	Mean Change (95% CI)	t	*p*-Value	Holm-Adjusted *p*-Value	Effect Size (d_z_)
Physical health	51.10 ± 18.58	56.21 ± 19.03	5.11 (1.29 to 8.92)	2.651	0.009	0.009	0.25
Psychological health	57.44 ± 18.35	67.27 ± 17.41	9.82 (6.15 to 13.49)	5.299	<0.001	<0.001	0.49
Social relationships	55.09 ± 17.47	62.00 ± 17.54	6.91 (3.47 to 10.35)	3.981	<0.001	<0.001	0.37
Environment	59.54 ± 17.55	65.12 ± 17.21	5.58 (2.13 to 9.03)	3.205	0.002	0.003	0.30

Note: Data are presented as mean ± SD. All comparisons were performed using paired-samples *t*-tests and included 117 complete pairs. Mean change was calculated as the post-amplification score minus the pre-amplification score; positive values indicate improvement. Effect sizes are reported as Cohen’s d_z_. SD, standard deviation; CI, confidence interval; WHOQOL-BREF, World Health Organization Quality of Life–BREF.

**Table 5:** Evaluation of communication and information before and after hearing amplification (N = 117).

Communication Item	Before Amplification, Mean ± SD	After Amplification, Mean ± SD
Do you experience that the doctor has been prepared for your interviews?	4.24 ± 0.96	4.29 ± 0.97
Do you experience that the nurses and nursing assistants have been prepared for your interviews?	3.95 ± 1.15	4.04 ± 1.11
Did the doctor use a language you could understand?	4.00 ± 1.17	4.30 ± 0.99
Did the nurses and nursing assistants use a language you could understand?	3.88 ± 1.21	4.18 ± 1.06
Have you been allowed to explain your problem/illness to the doctor?	3.98 ± 1.20	4.09 ± 1.22
Have you been allowed to explain your problem/illness to the nurses and nursing assistants?	3.85 ± 1.13	3.97 ± 1.17
Did the doctor explain to you about examinations and treatments?	3.93 ± 1.17	4.15 ± 1.16
Did the nurses and nursing assistants explain to you about examinations and treatments?	3.72 ± 1.24	4.06 ± 1.18
Did the doctor explain to you about plans?	3.94 ± 1.18	4.20 ± 1.09
Did the nurses and nursing assistants explain to you about plans?	3.69 ± 1.27	4.04 ± 1.16
Are you satisfied with the information you received from the doctor?	4.01 ± 1.06	4.38 ± 0.95
Are you satisfied with the information you received from the nurses and nursing assistants?	3.74 ± 1.15	4.13 ± 1.08

Note: Data are presented as mean ± standard deviation (SD). Individual questionnaire items are presented descriptively and were not subjected to inferential pre–post comparisons. Higher scores indicate greater satisfaction with communication and information.

**Table 6:** Associations between total IOI-HA score and quality of life following hearing amplification.

Variable	WHOQOL-BREF Domain	Post-Amplification Score	Change Score (Δ)
Total IOI-HA score	Physical health	0.222 *	0.236 *
	Psychological health	0.257 **	0.217 *
	Social relationships	0.304 **	0.275 **
	Environment	0.323 ***	0.170

Note: Values represent Spearman’s rank correlation coefficients (ρ). The total IOI-HA score was correlated with WHOQOL-BREF domain scores after hearing amplification and with changes (Δ) in WHOQOL-BREF domain scores. Change scores were calculated as post-amplification minus pre-amplification scores, with positive values indicating improvement in quality of life. Higher total IOI-HA scores indicate better self-reported hearing aid outcomes. *p* < 0.05 (*), *p* < 0.01 (**), and *p* < 0.001 (***). IOI-HA, International Outcome Inventory for Hearing Aids; WHOQOL-BREF, World Health Organisation Quality of Life-BREF.

**Table 7:** Associations between post-amplification communication satisfaction and quality of life.

Variable	WHOQOL-BREF Domain	Post-Amplification Score
Post-amplification total communication satisfaction score	Physical health	0.274 **
	Psychological health	0.247 **
	Social relationships	0.399 ***
	Environment	0.344 ***

Note: Values represent Spearman’s rank correlation coefficients (ρ). The total communication satisfaction score measured after hearing amplification was correlated with post-amplification WHOQOL-BREF domain scores. Higher communication satisfaction scores indicate greater satisfaction with communication with healthcare professionals after hearing amplification. *p* < 0.01 (**), and *p* < 0.001 (***). WHOQOL-BREF, World Health Organisation Quality of Life-BREF.

**Table 8:** Associations between changes in communication satisfaction and changes in quality of life following hearing amplification.

Variable	WHOQOL-BREF Domain	Change Score (Δ), ρ
Change in total communication satisfaction score (Δ)	Physical health	0.038
	Psychological health	0.072
	Social relationships	0.127
	Environment	0.116

Note: Values represent Spearman’s rank correlation coefficients (ρ). Correlations were calculated between changes (Δ) in the total communication satisfaction score and changes (Δ) in WHOQOL-BREF domain scores. All change scores were calculated as post-amplification minus pre-amplification scores, with positive values indicating an increase in the respective score. None of the observed correlations reached statistical significance (*p* > 0.05). WHOQOL-BREF, World Health Organisation Quality of Life-BREF.

**Table 9:** Hierarchical linear regression analysis of factors associated with physical health after hearing amplification (N = 117).

Variable	Model 1 (Control Variables)							Model 2 (Control Variables + Variables of Interest)				
	B	β	t	*p*	VIF	95% CI for B	B	β	t	*p*	VIF	95% CI for B
Baseline physical health score	0.338	0.33	3.66	<0.001	1.27	0.155 to 0.522	0.348	0.34	4.07	<0.001	1.27	0.179 to 0.517
Gender	−12.127	−0.32	−3.82	<0.001	1.09	−18.422 to −5.831	−11.014	−0.29	−3.73	<0.001	1.11	−16.874 to −5.153
Age	0.457	0.16	1.88	0.063	1.06	−0.026 to 0.940	0.529	0.18	2.35	0.021	1.07	0.082 to 0.975
Chronic disease/comorbidity	−7.292	−0.16	−1.98	0.050	1.01	−14.596 to 0.011	−9.014	−0.20 *	−2.62	0.010	1.04	−15.827 to −2.201
Baseline HHIE total score	0.017	0.02	0.24	0.809	1.23	−0.121 to 0.154	0.035	0.05	0.54	0.588	1.27	−0.093 to 0.164
IOI-HA total score	–	–	–		–	–	1.031	0.31	3.92	<0.001	1.14	0.510 to 1.552
Post-amplification communication satisfaction score	–	–	–		–	–	0.152	0.09	1.14	0.259	1.19	−0.114 to 0.418
R^2^			0.284						0.403			
Adjusted R^2^			0.252						0.364			
F			8.83 ***						10.49 ***			
ΔR^2^			–						0.118 ***			
F change			–						10.77 ***			

Note: The dependent variable was the WHOQOL-BREF physical health domain score after hearing amplification. Model 1 included the baseline physical health score, gender, age, chronic disease/comorbidity and baseline total HHIE score. Model 2 additionally included the IOI-HA total score and post-amplification communication satisfaction score. Male gender and absence of chronic disease/comorbidity were used as the reference categories. β, standardised regression coefficient; CI, confidence interval; VIF, variance inflation factor; HHIE, Hearing Handicap Inventory for the Elderly; IOI-HA, International Outcome Inventory for Hearing Aids. Holm adjustment was applied separately across the four WHOQOL-BREF domain-specific tests of the IOI-HA and post-amplification communication satisfaction coefficients. * *p* < 0.05, *** *p* < 0.001.

**Table 10:** Hierarchical linear regression analysis of factors associated with psychological health after hearing amplification (N = 117).

Variable	Model 1 (Control Variables)						Model 2 (Control Variables + Variables of Interest)					
	B	β	t	*p*	VIF	95% CI for B	B	β	t	*p*	VIF	95% CI for B
Baseline psychological health score	0.327	0.35	3.94	<0.001	1.23	0.163 to 0.492	0.311	0.33	4.15	<0.001	1.23	0.162 to 0.459
Gender	−10.926	−0.32	−3.78	<0.001	1.11	−16.653 to −5.199	−9.945	−0.29	−3.78	<0.001	1.13	−15.162 to −4.728
Age	0.572	0.21	2.61	0.010	1.06	0.138 to 1.007	0.643	0.24	3.24	0.002	1.07	0.250 to 1.036
Chronic disease/comorbidity	−7.050	−0.17	−2.12	0.036	1.02	−13.640 to −0.461	−8.797	−0.21	−2.90	0.005	1.05	−14.817 to −2.778
Baseline HHIE total score	0.110	0.15	1.81	0.073	1.16	−0.011 to 0.230	0.122	0.17 *	2.19	0.031	1.21	0.011 to 0.233
IOI-HA total score	–	–	–	–	–	–	1.007	0.33	4.35	<0.001	1.14	0.548 to 1.465
Post-amplification communication satisfaction score	–	–	–	–	–	–	0.168	0.11	1.43	0.156	1.18	−0.065 to 0.402
R^2^			0.307						0.447			
Adjusted R^2^			0.276						0.411			
F			9.84 ***						12.56 ***			
ΔR^2^			–						0.139 ***			
F change			–						13.73 ***			

Note: The dependent variable was the WHOQOL-BREF psychological health domain score after hearing amplification. Model 1 included the baseline psychological health score, gender, age, chronic disease/comorbidity, and baseline total HHIE score. Model 2 additionally included the IOI-HA total score and post-amplification communication satisfaction score. Male gender and absence of chronic disease/comorbidity were used as the reference categories. β, standardised regression coefficient; CI, confidence interval; VIF, variance inflation factor; HHIE, Hearing Handicap Inventory for the Elderly; IOI-HA, International Outcome Inventory for Hearing Aids. Holm adjustment was applied separately across the four WHOQOL-BREF domain-specific tests of the IOI-HA and post-amplification communication satisfaction coefficients. * *p* < 0.05, *** *p* < 0.001.

**Table 11:** Hierarchical linear regression analysis of factors associated with social relationships domain scores after hearing amplification (N = 117).

Variable	Model 1 (Control Variables)						Model 2 (Control Variables + Variables of Interest)					
	B	β	t	*p*	VIF	95% CI for B	B	β	t	*p*	VIF	95% CI for B
Baseline social relationships score	0.425	0.42	4.88	<0.001	1.10	0.252 to 0.598	0.378	0.38	4.74	<0.001	1.15	0.220 to 0.535
Gender	−6.213	−0.18	−2.07	0.040	1.07	−12.150 to −0.277	−4.788	−0.14	−1.77	0.079	1.09	−10.144 to 0.568
Age	0.575	0.21	2.48	0.015	1.06	0.116 to 1.034	0.637	0.24	3.07	0.003	1.07	0.225 to 1.049
Chronic disease/comorbidity	0.256	0.01	0.07	0.942	1.01	−6.675 to 7.188	−1.148	−0.03	−0.36	0.718	1.04	−7.424 to 5.127
Baseline HHIE total score	0.023	0.03	0.38	0.707	1.09	−0.099 to 0.146	0.046	0.07	0.83	0.409	1.11	−0.065 to 0.158
IOI-HA total score		–	–		–	–	0.882	0.29	3.64	<0.001	1.14	0.402 to 1.363
Post-amplification communication satisfaction score		–	–		–	–	0.339	0.22	2.69	0.008	1.24	0.089 to 0.589
R^2^			0.240						0.403			
Adjusted R^2^			0.206						0.364			
F			7.03 ***						10.49 ***			
ΔR^2^			–						0.162 ***			
F change			–						14.78 ***			

Note: The dependent variable was the WHOQOL-BREF social relationships domain score after hearing amplification. Model 1 included the baseline social relationships score, gender, age, chronic disease/comorbidity and baseline total HHIE score. Model 2 additionally included the IOI-HA total score and post-amplification communication satisfaction score. Male gender and absence of chronic disease/comorbidity were used as the reference categories. β, standardised regression coefficient; CI, confidence interval; VIF, variance inflation factor; HHIE, Hearing Handicap Inventory for the Elderly; IOI-HA, International Outcome Inventory for Hearing Aids. Holm adjustment was applied separately across the four WHOQOL-BREF domain-specific tests of the IOI-HA and post-amplification communication satisfaction coefficients. The association between post-amplification communication satisfaction and the social relationships domain remained statistically significant after Holm adjustment (adjusted *p* = 0.032). *** *p* < 0.001.

**Table 12:** Hierarchical linear regression analysis of factors associated with the environment domain after hearing amplification (N = 117).

Variable	Model 1 (Control Variables)						Model 2 (Control Variables + Variables of Interest)					
	B	β	t	*p*	VIF	95% CI for B	B	β	t	*p*	VIF	95% CI for B
Baseline environment score	0.397	0.41	4.57	<0.001	1.14	0.225 to 0.570	0.340	0.35	4.12	<0.001	1.19	0.176 to 0.503
Gender	−4.117	−0.12	−1.39	0.167	1.08	−9.982 to 1.747	−3.221	−0.09	−1.16	0.247	1.09	−8.703 to 2.262
Age	0.652	0.25	2.85	0.005	1.07	0.199 to 1.105	0.726	0.27	3.41	0.001	1.07	0.304 to 1.148
Chronic disease/comorbidity	0.167	0.00	0.05	0.961	1.01	−6.661 to 6.995	−1.171	−0.03	−0.36	0.718	1.04	−7.584 to 5.241
Baseline HHIE total score	0.042	0.06	0.68	0.496	1.12	−0.081 to 0.165	0.049	0.07	0.84	0.404	1.15	−0.067 to 0.164
IOI-HA total score	–	–	–	–	–	–	0.873	0.29	3.52	0.001	1.14	0.382 to 1.364
Post-amplification communication satisfaction score	–	–	–	–	–	–	0.201	0.13	1.57	0.120	1.22	−0.053 to 0.454
R^2^			0.233						0.352			
Adjusted R^2^			0.199						0.310			
F			6.76 ***						8.46 ***			
ΔR^2^			–						0.119 ***			
F change			–						9.97 ***			

Note: The dependent variable was the WHOQOL-BREF environment domain score after hearing amplification. Model 1 included the baseline environment score, gender, age, chronic disease/comorbidity, and baseline total HHIE score. Model 2 additionally included the IOI-HA total score and post-amplification communication satisfaction score. Male gender and absence of chronic disease/comorbidity were used as the reference categories. β, standardised regression coefficient; CI, confidence interval; VIF, variance inflation factor; HHIE, Hearing Handicap Inventory for the Elderly; IOI-HA, International Outcome Inventory for Hearing Aids. Holm adjustment was applied separately across the four WHOQOL-BREF domain-specific tests of the IOI-HA and post-amplification communication satisfaction coefficients. *** *p* < 0.001.

## Data Availability

The original contributions presented in the study are included in the article; further inquiries can be directed to the corresponding author.
